# Supplementary material for: Reaction of Corroles with Sarcosine and Paraformaldehyde: A New Facet of Corrole Chemistry
Source: Int J Mol Sci. 2022 Nov 5;23(21):13581. doi: 10.3390/ijms232113581 (PMC9658120; doi:10.3390/ijms232113581)
Supplement: Supplementary file 1 [file ijms-23-13581-s001.zip › ijms-1874454-supplementary.pdf]

Reaction of corroles with sarcosine and paraformaldehyde: a new facet on corrole chemistry

Joana F. B. Barata [1,2,*], Paula S. S. Lacerda [1,2], Maria Graça P. M. S. Neves [2,*], José A. S. Cavaleiro [2], Catarina I. V. Ramos [2], Augusto C. Tomé [2], Paulo E. Abreu [3] and Alberto A. C. C. Pais [3]

1 CESAM, University of Aveiro, 3810-193 Aveiro, Portugal

2 LAQV-REQUIMTE, Department of Chemistry, University of Aveiro, 3810-193 Aveiro, Portugal

3 Department of Chemistry, University of Coimbra, 3004-535 Coimbra, Portugal

* Correspondence: jbarata@ua.pt (J.F.B.B.); gneves@ua.pt (M.G.M.P.S.N.)

Contents

1. NMR spectra of compound 4 and compound 5

Figure S1 - ^{1}H NMR spectrum of compound **4** in $CDCl_3$ + pyridine-*d*$_5$.

Figure S2 – Partial COSY (^{1}H/^{1}H) spectrum of compound **4** in $CDCl_3$ + pyridine-*d*$_5$.

Figure S3 – NOESY (^{1}H/^{1}H) spectrum of compound **4** in $CDCl_3$ + pyridine-*d*$_5$.

Figure S4 – HSQC (^{1}H/^{13}C) spectrum of compound **4** in $CDCl_3$ + pyridine-*d*$_5$.

Figure S5 - HMBC (^{1}H/^{13}C) spectrum of compound **4** in $CDCl_3$ + pyridine-*d*$_5$.

Figure S6 - ^{1}H NMR spectrum of compound **5** in $CDCl_3$ + pyridine-*d*$_5$.

Figure S7 – Partial COSY (^{1}H/^{1}H) spectrum of compound **5** in $CDCl_3$ + pyridine-*d*$_5$.

Figure S8 - Partial NOESY (^{1}H/^{1}H) spectrum of compound **5** in $CDCl_3$ + pyridine-*d*$_5$.

Figure S9 – HSQC (^{1}H/^{13}C) spectrum of compound **5** in $CDCl_3$ + pyridine-*d*$_5$.

Figure S10 - HMBC (^{1}H/^{13}C) spectrum of compound **5** in $CDCl_3$ + pyridine-*d*$_5$.

2. NMR spectra of compound 9

Figure S11 - ^{1}H NMR spectrum of compound **9** in CD_3COCD_3.

Figure S12 – ^{1}H NMR spectrum of compound **9** at 10 °C in CD_3COCD_3.

Figure S13 – ^{13}C NMR spectrum of compound **9** in CD_3COCD_3

Figure S14 – HSQC ($^{1}H/^{13}C$) spectrum of compound **9** in CD_3COCD_3.

Figure S15 – HMBC ($^{1}H/^{13}C$) spectrum of compound **9** in CD_3COCD_3.

3. Mass Spectra of compound 9

Figure S16 – Mass spectrum of compound **9** in the range of m/z 740-1000

Figure S17 – Product-ion spectrum of compound **9**

Figure S18 – HRMS-ESI(+) spectrum of compound **9**

4. Enthalpy PM7 semi-empirical calculations

Table S1 – Calculations of the formation enthalpies for precursor **8**

Table S2 – Structures and calculations of formation enthalpies for compound **9** tautomers (**9T1**, **9T2** and **9T3**) and structure for tautomer **9T1** optimized with MOPAC2016.

5. Spectophotometric interactions

Figure S19 – Absorption spectra of behaviour of corroles **8** and **9** in the presence of **(A)** tetrabutylammonium fluoride (TBAF) and **(B)** acetic acid (AcOH) and AcOH plus triethylamine (TEA).

1. NMR spectra of compound 4 and compound 5

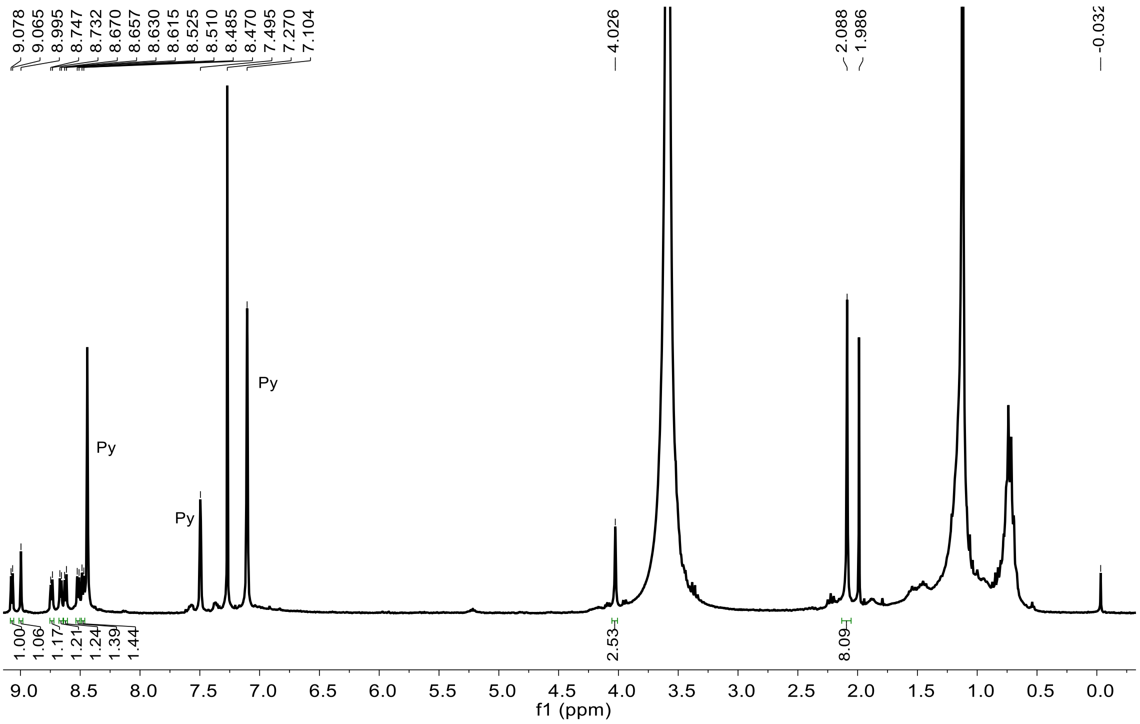

Figure S1 - ^{1}H NMR spectrum of compound **4** in $CDCl_3$ + pyridine-*d*$_5$.

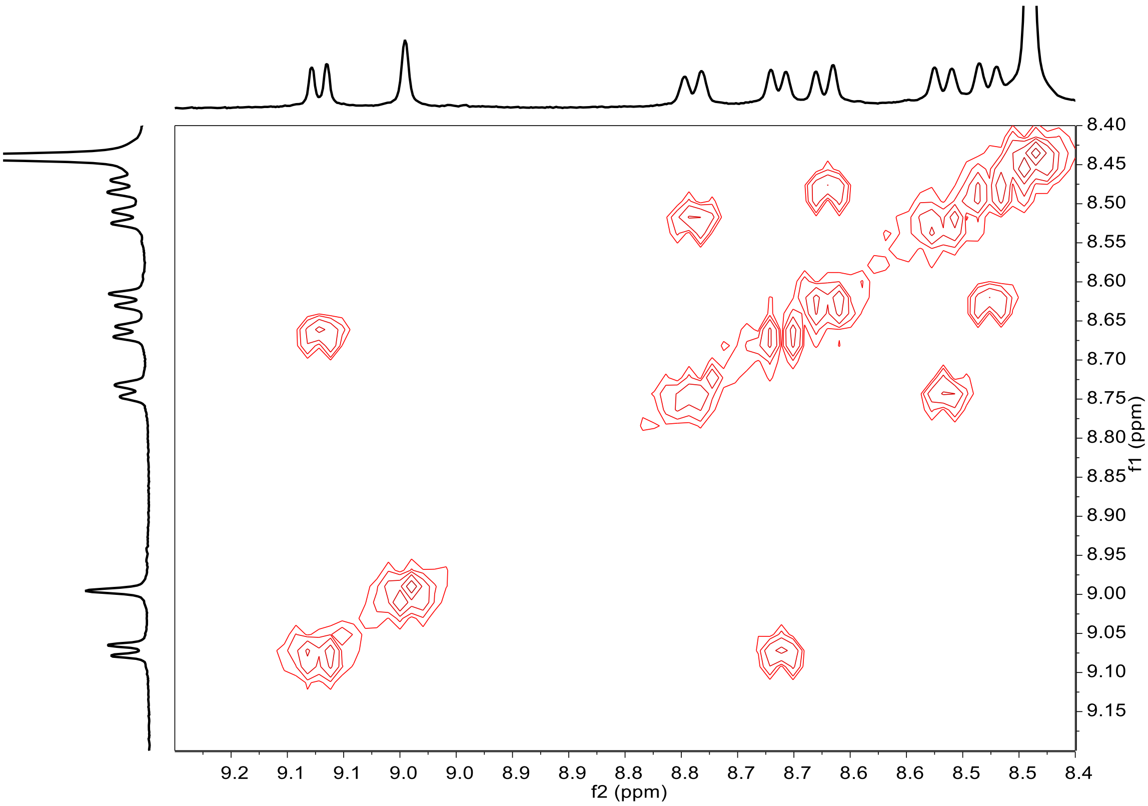

Figure S2 – Partial COSY (^{1}H/^{1}H) spectrum of compound **4** in $CDCl_3$ + pyridine-*d*$_5$.

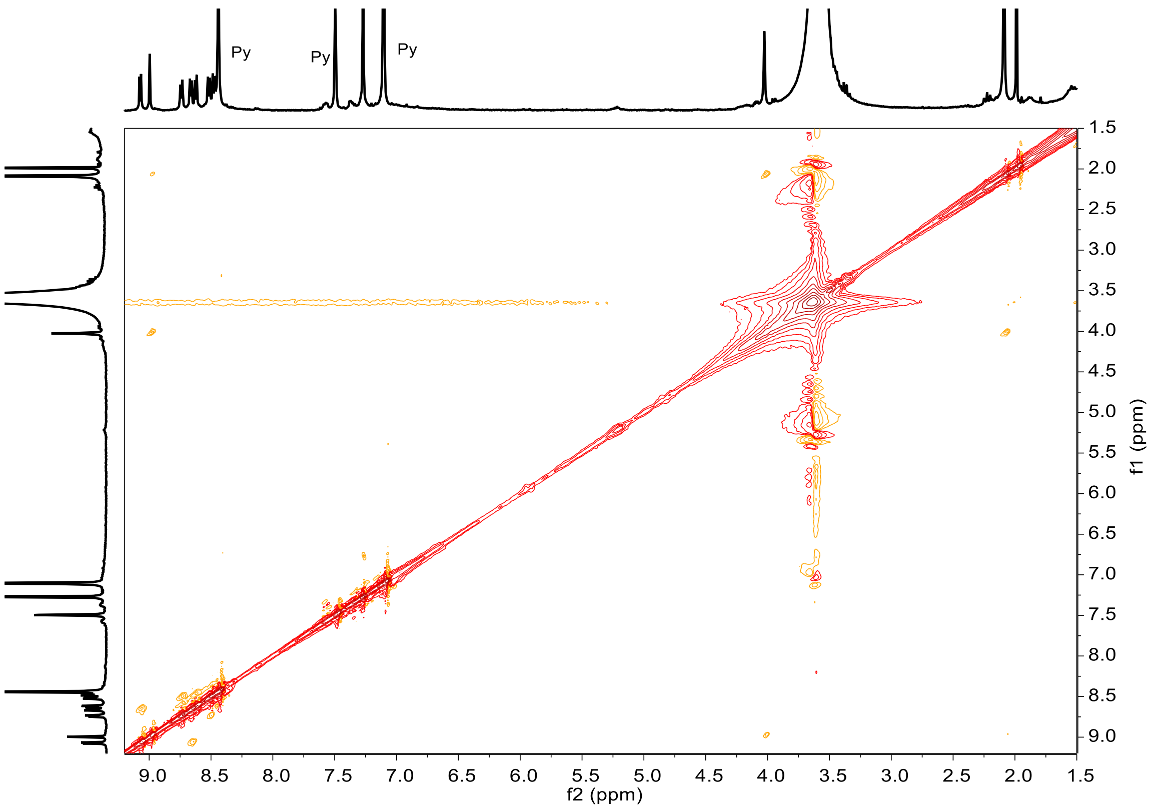

Figure S3 – NOESY ($^{1}H/^{1}H$) spectrum of compound **4** in $CDCl_3$ + pyridine-*d_5*.

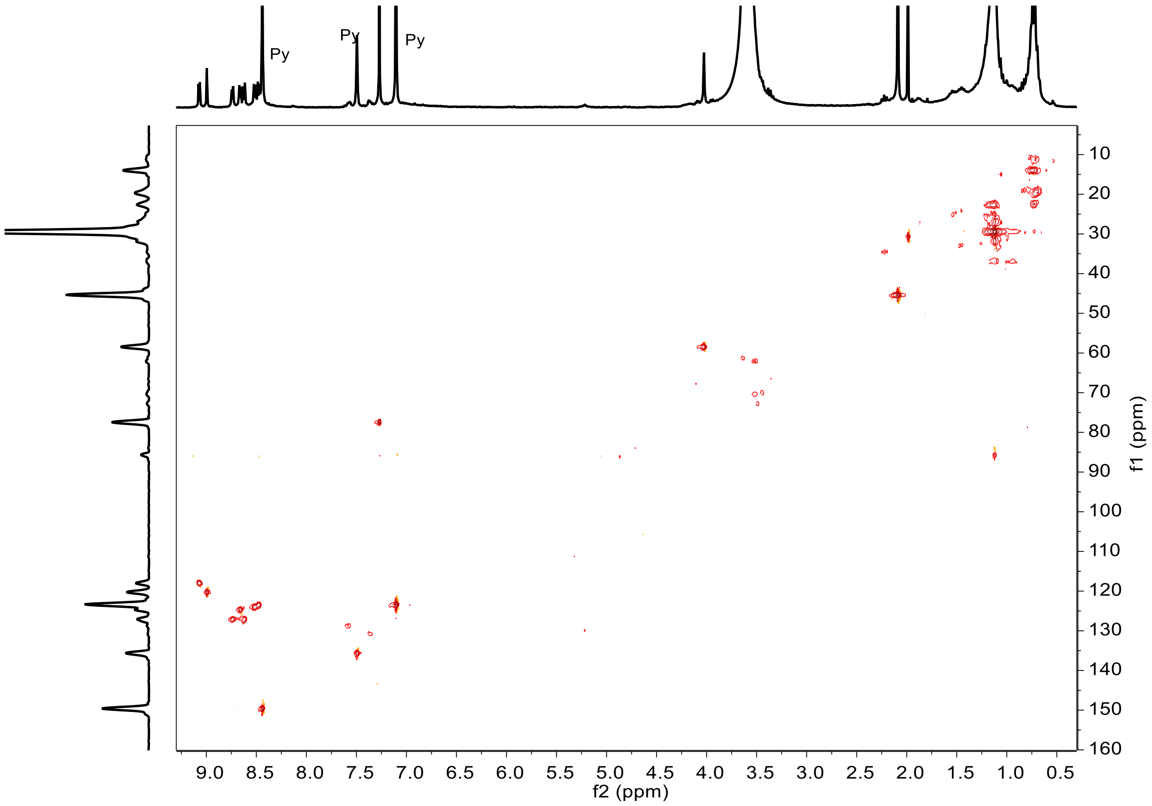

Figure S4 – HSQC ($^{1}H/^{13}C$) spectrum of compound **4** in $CDCl_3$ + pyridine-*d_5*.

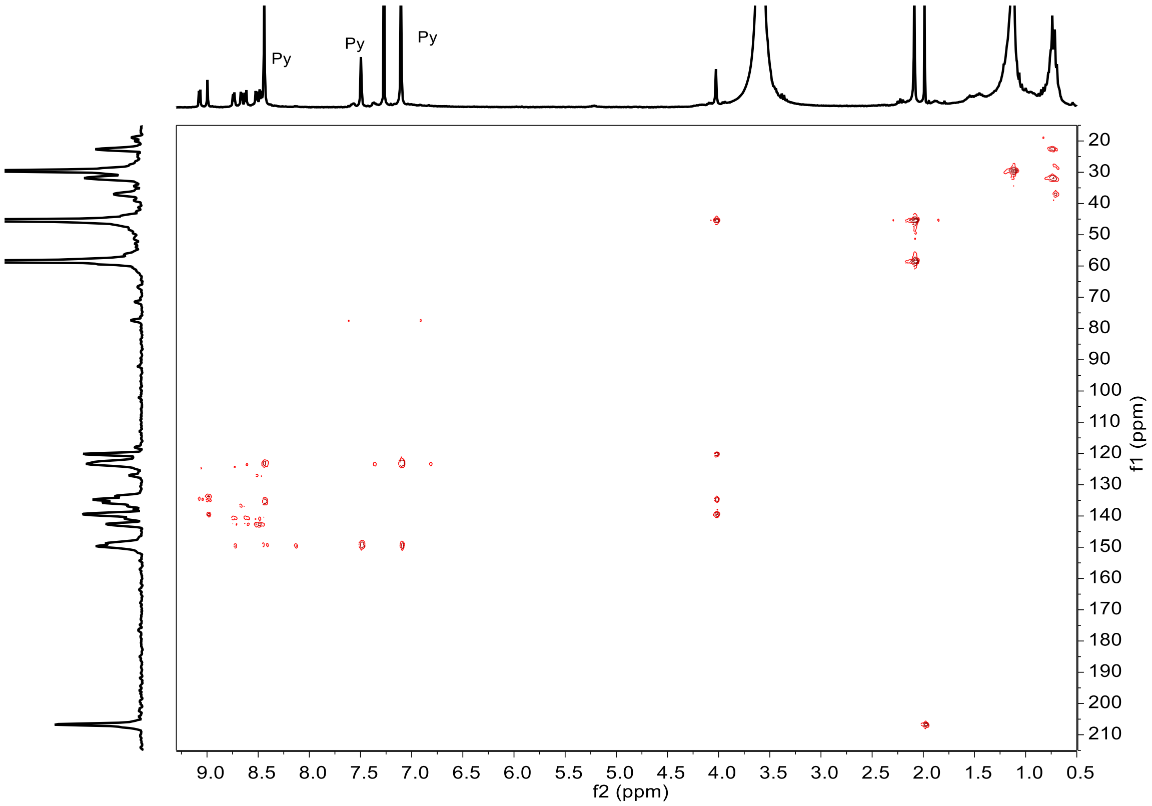

Figure S5 - HMBC ($^{1}H/^{13}C$) spectrum of compound **4** in $CDCl_3$ + pyridine-d_5.

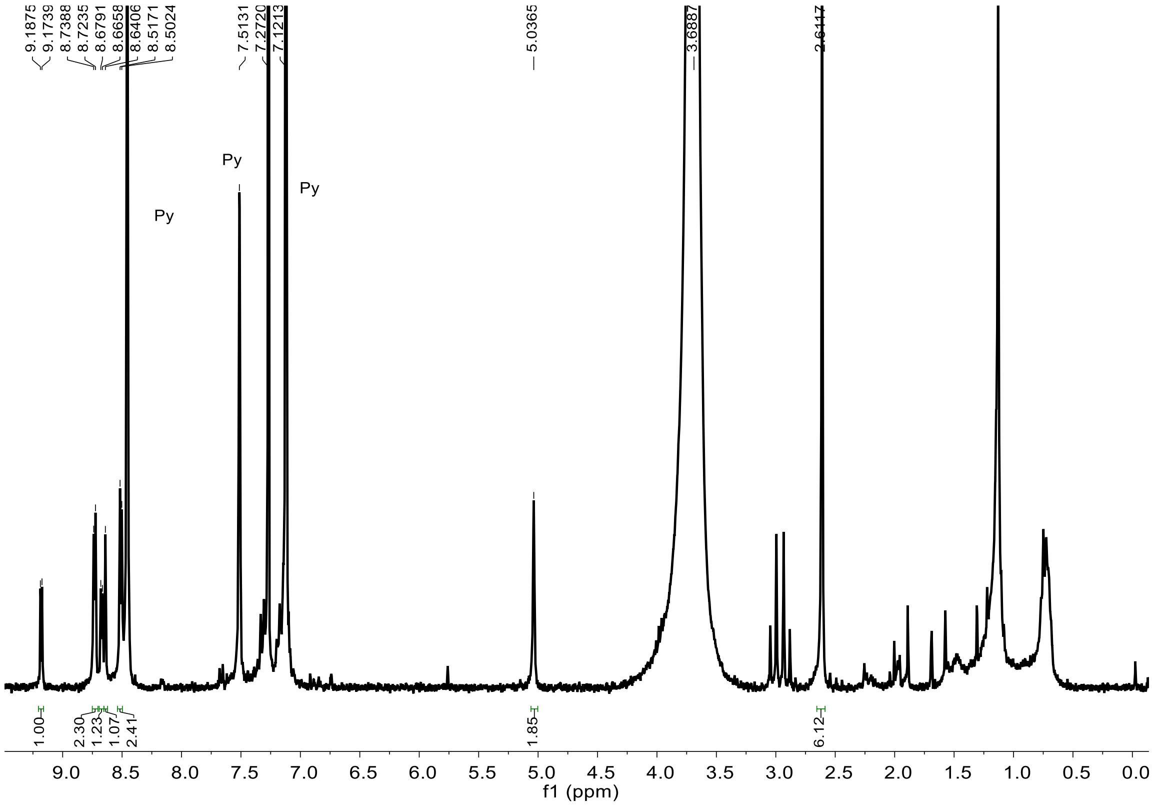

Figure S6 - ^{1}H NMR spectrum of compound **5** in $CDCl_3$ + pyridine-d_5.

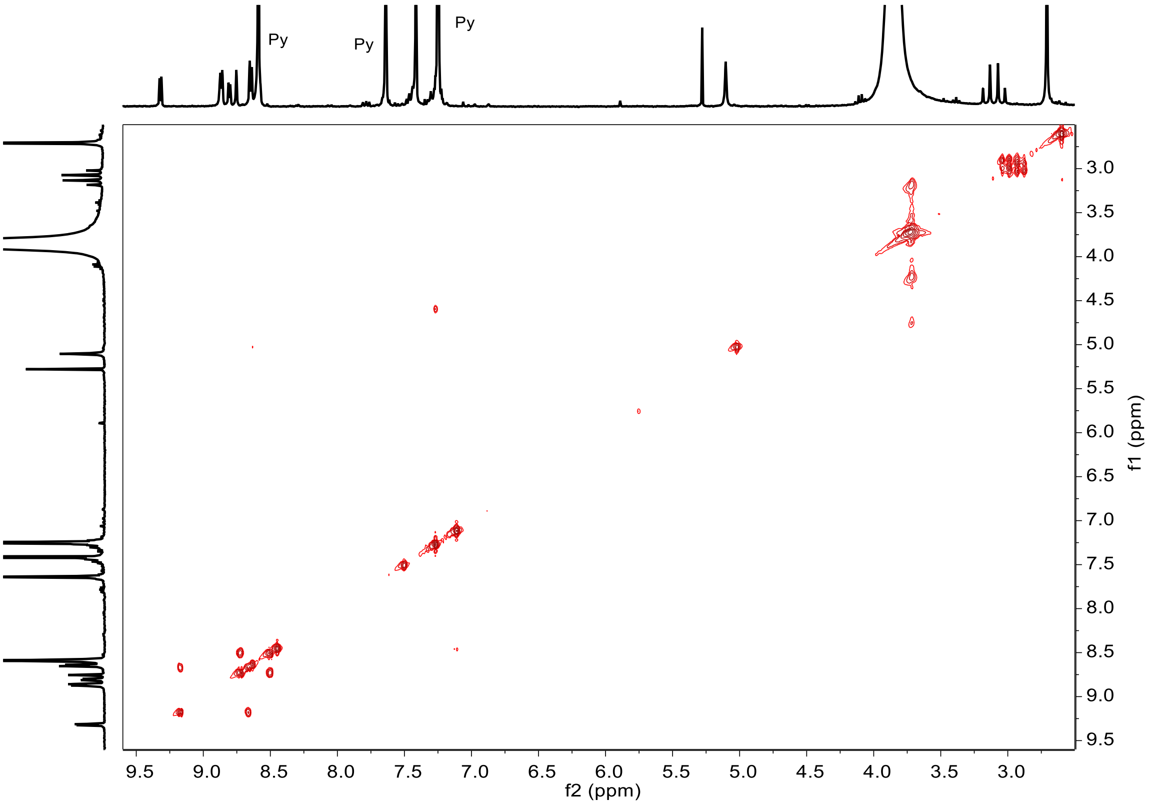

Figure S7 – Partial COSY ($^1H/^1H$) spectrum of compound **5** in $CDCl_3$ + pyridine-d_5.

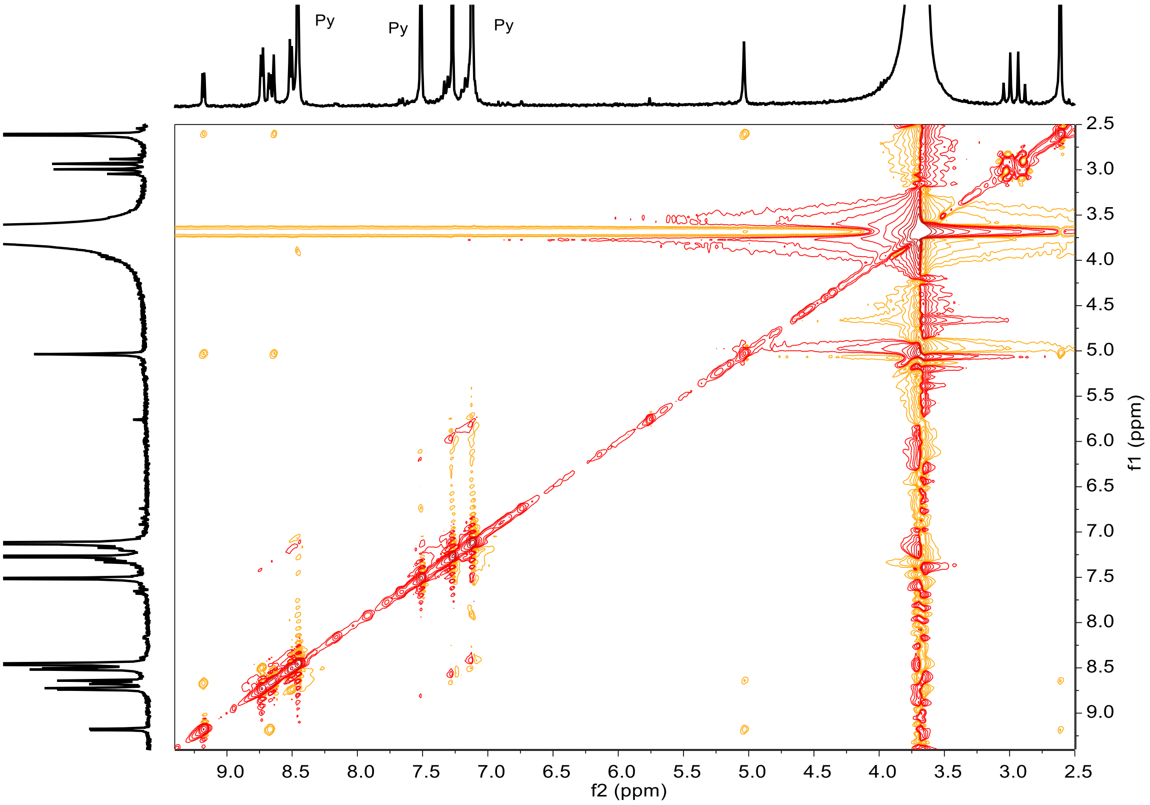

Figure S8 - Partial NOESY ($^1H/^1H$) spectrum of compound **5** in $CDCl_3$ + pyridine-d_5.

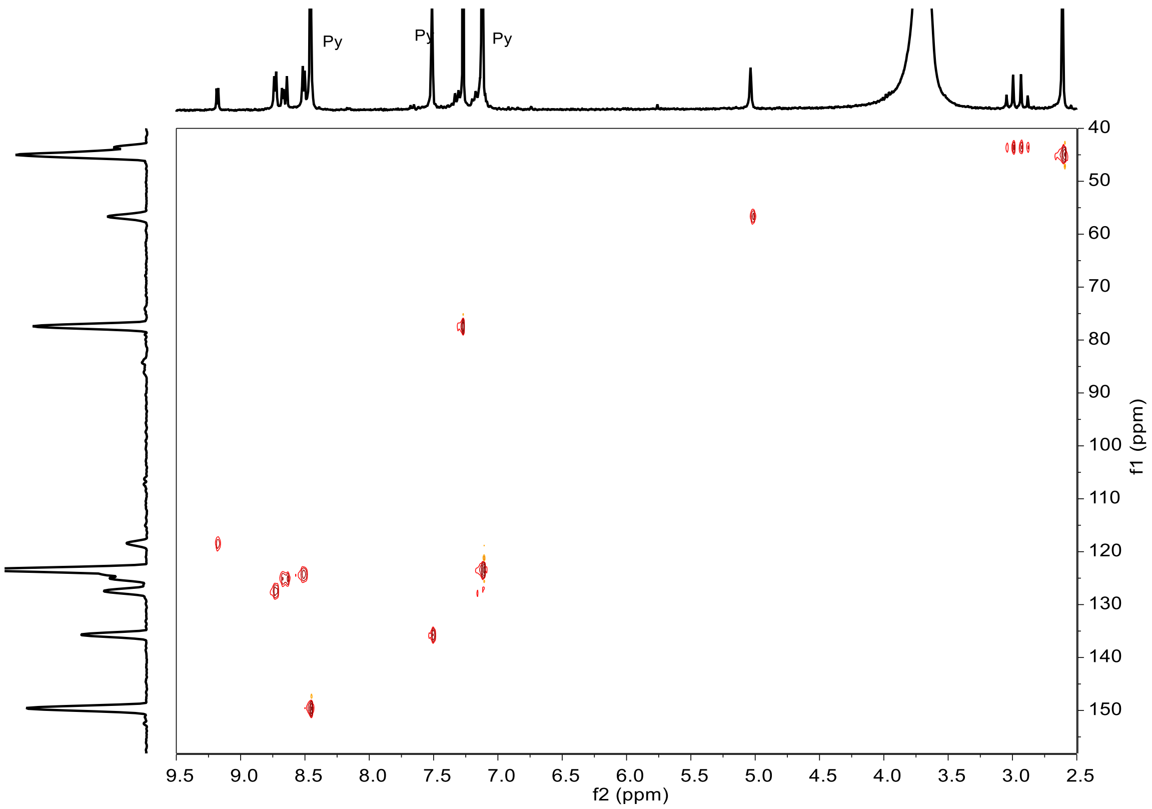

Figure S9 – HSQC (^{1}H/^{13}C) spectrum of compound **5** in $CDCl_3$ + pyridine-*d*$_5$.

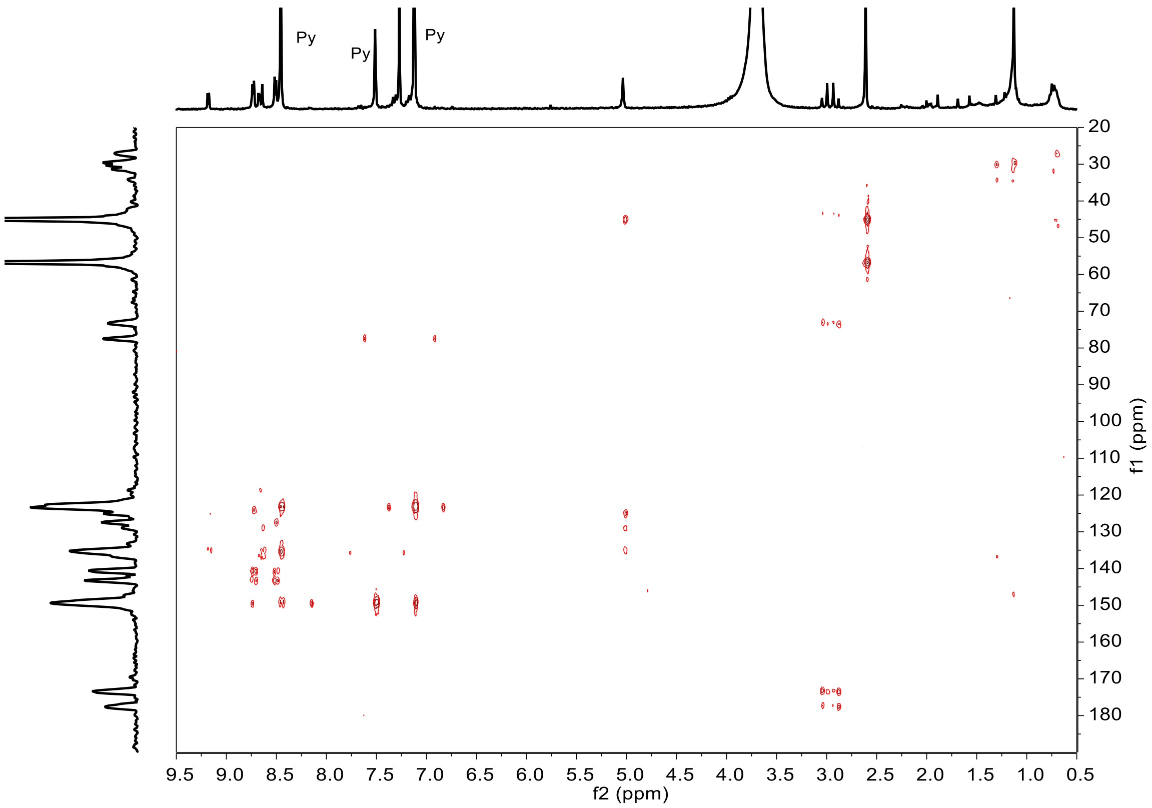

Figure S10 - HMBC (^{1}H/^{13}C) spectrum of compound **5** in $CDCl_3$ + pyridine-*d*$_5$.

2. NMR spectra of compound 9

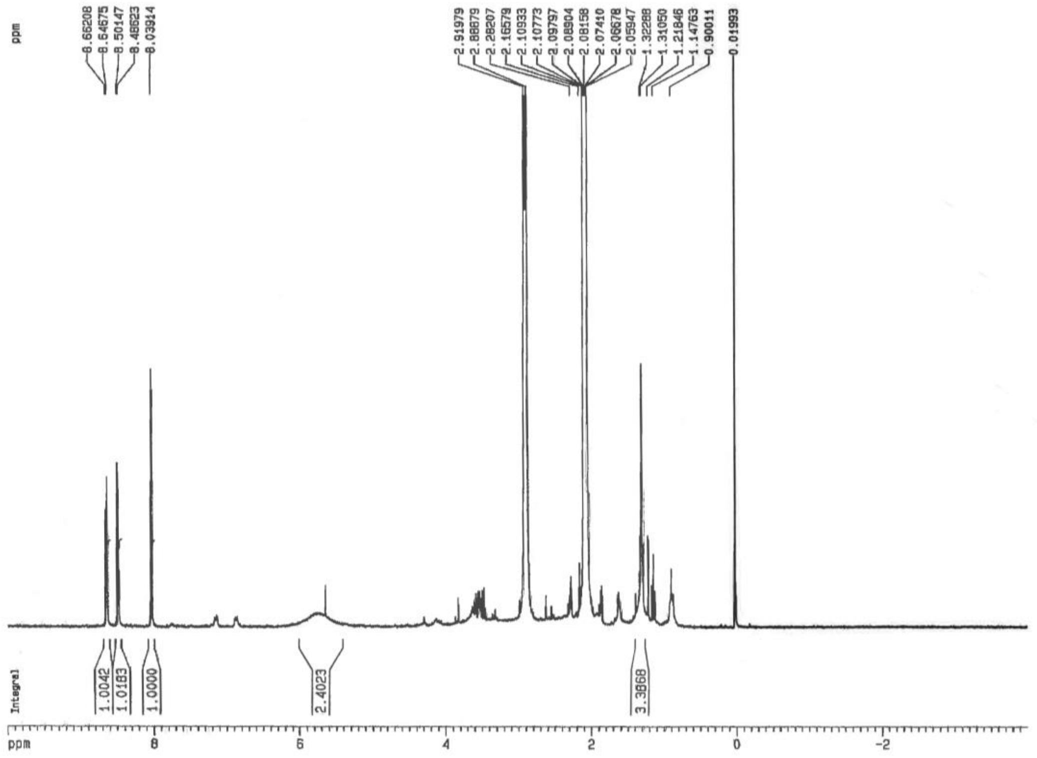

Figure S11 - ^{1}H NMR spectrum of compound **9** in CD_3COCD_3.

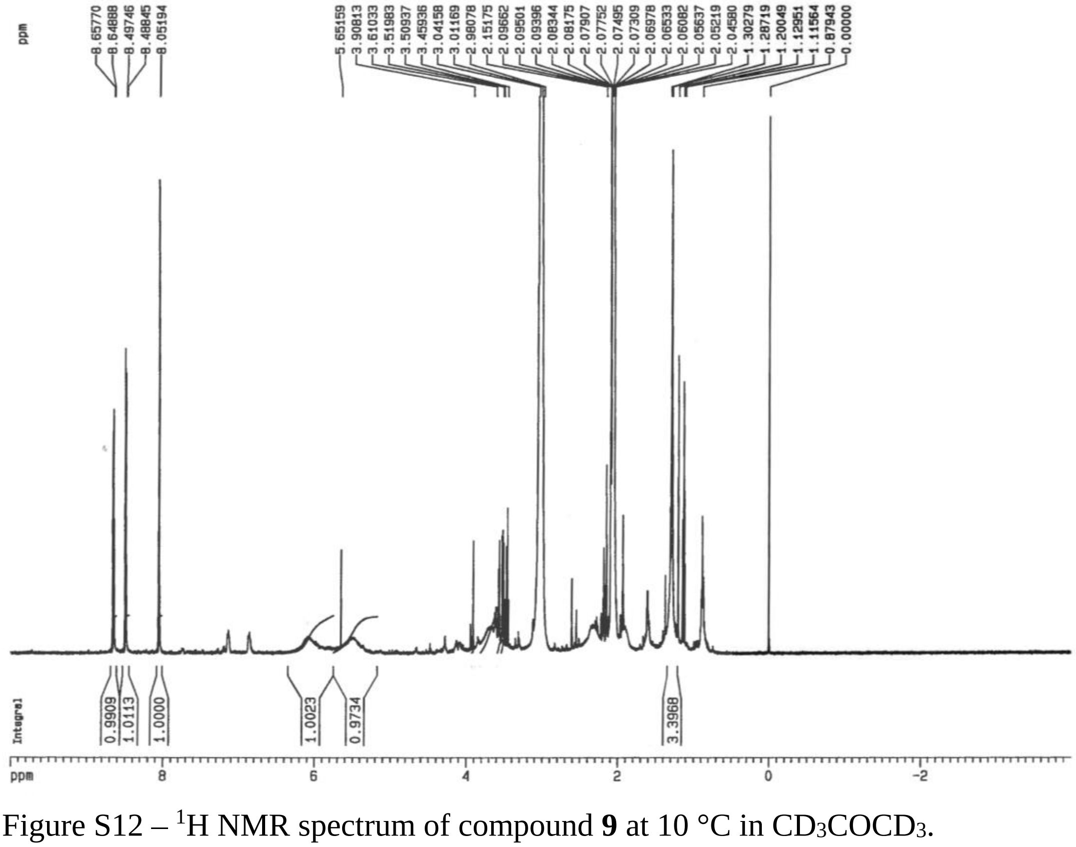

Figure S12 – ^{1}H NMR spectrum of compound **9** at 10 °C in CD_3COCD_3.

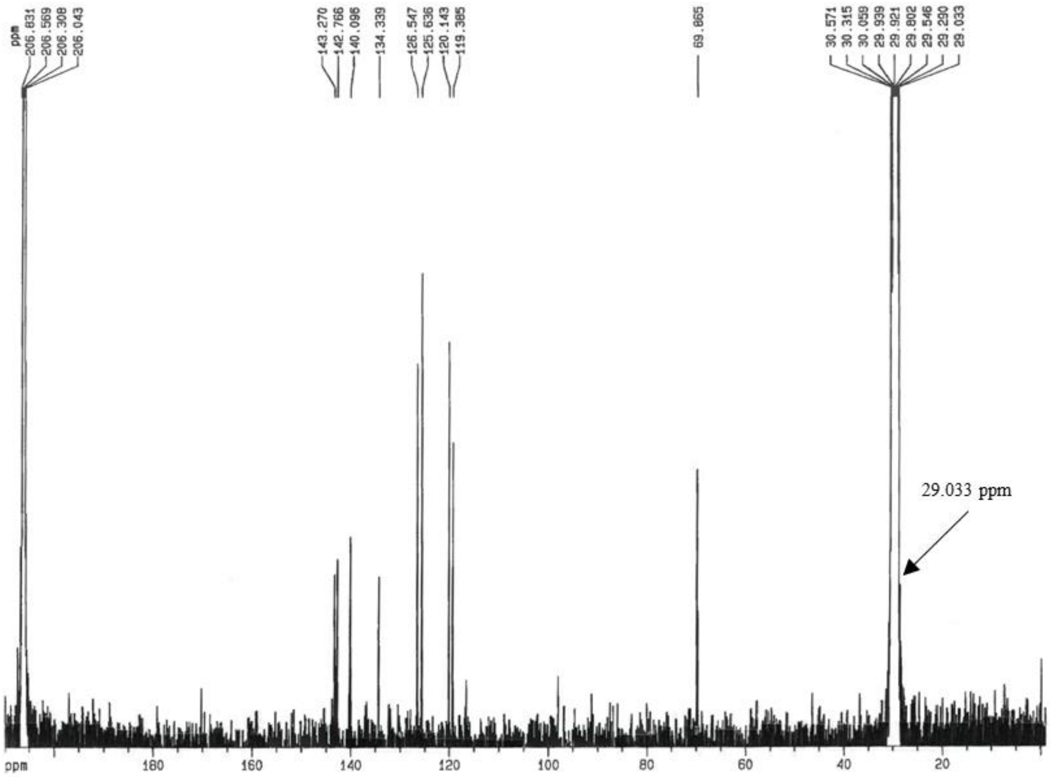

Figure S13 – ^{13}C NMR spectrum of compound **9** in CD_3COCD_3.

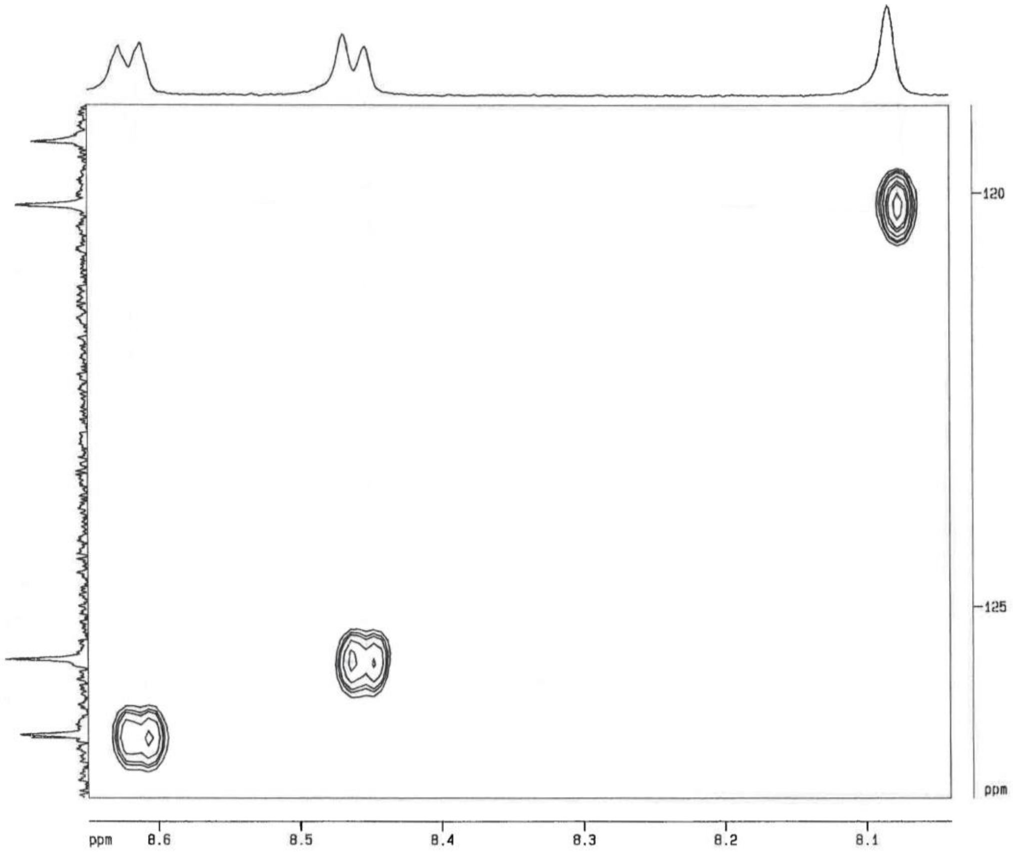

Figure S14 – HSQC (^{1}H/^{13}C) spectrum of compound **9** in CD_3COCD_3.

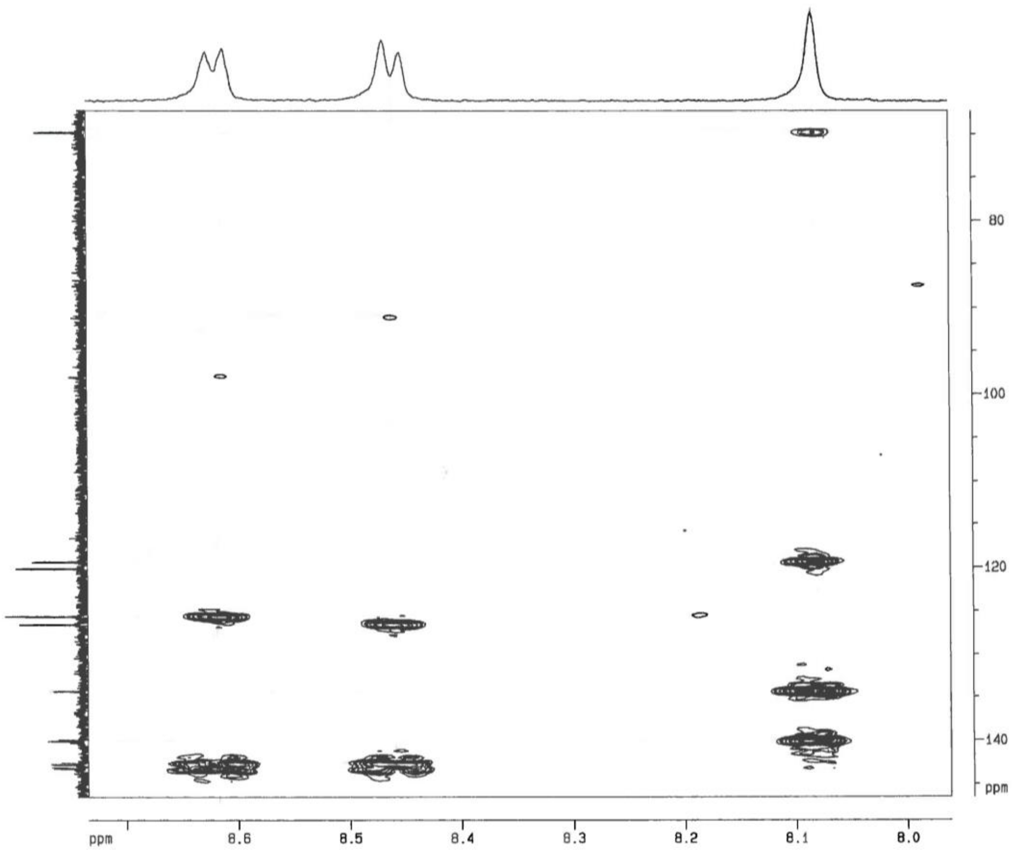

Figure S15– HMBC ($^1H/^{13}C$) spectrum of compound **9** in CD_3COCD_3.

3. Mass Spectra of compound 9

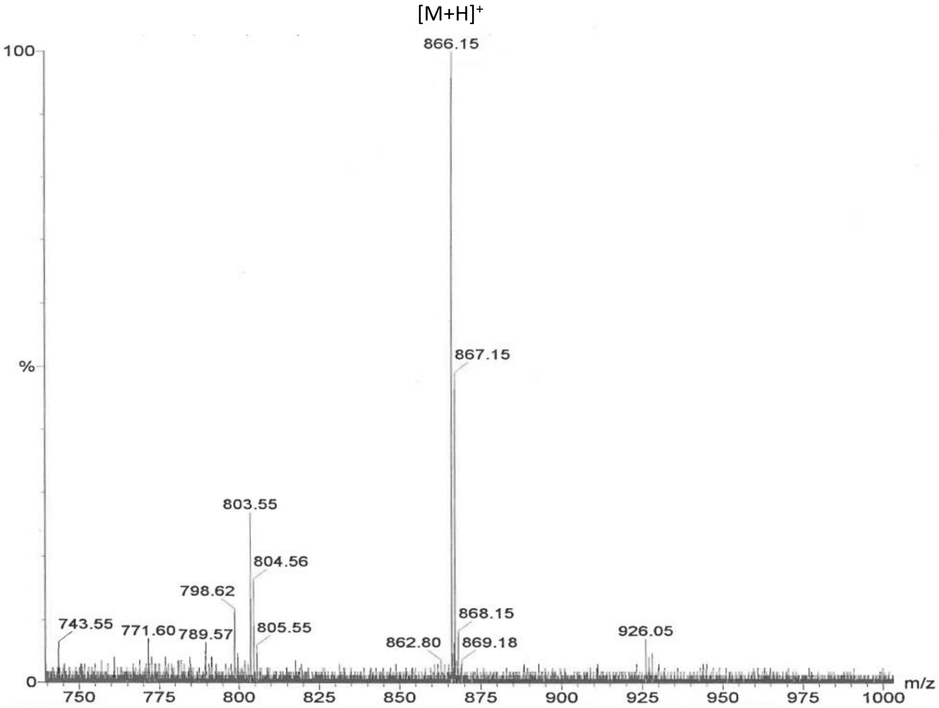

Figure S16 – Mass spectrum of compound **9** in the range of m/z 740-1000

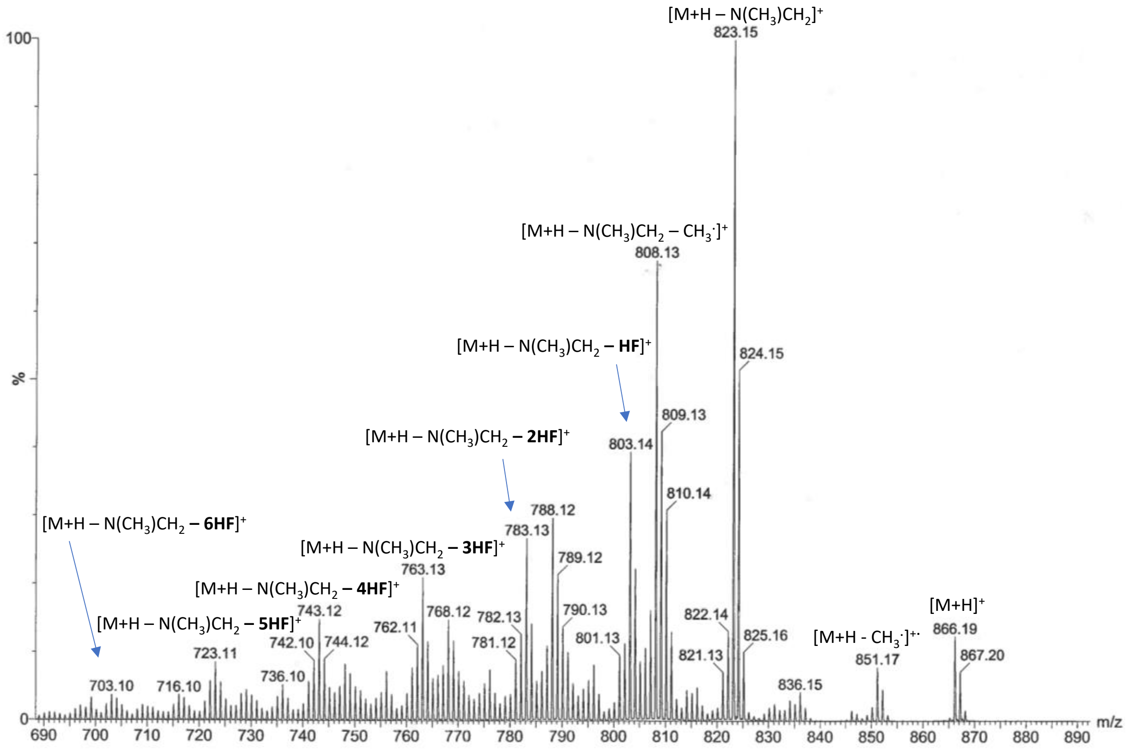

Figure S17 – Product-ion spectrum of compound **9**

Acquisition Parameter
Capillary Exit voltage for ESI 300.000000 V

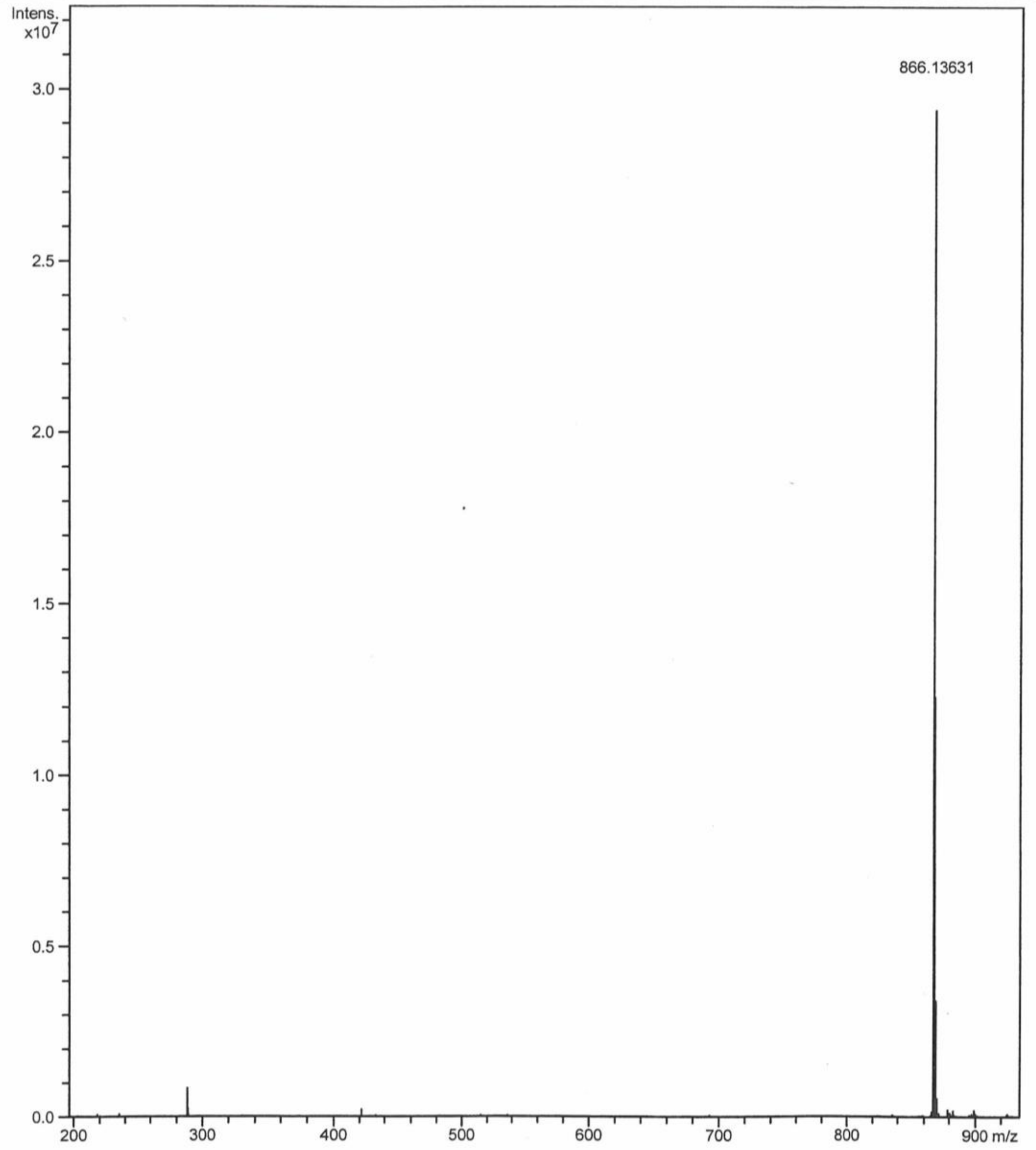

Mass Spectrum Molecular Formula Report

Sum Formula	Sigma	m/z	Err [ppm]	Mean Err [ppm]	Err [mDa]	rdb	N Rule	e⁻
C 41 H 19 F 15 N 5	0.031	866.13955	3.74	3.21	3.24	27.50	ok	even
C 46 H 21 F 9 N 3 O 5	0.056	866.13320	-3.59	-3.99	-3.11	33.50	ok	even
C 49 H 20 F 8 N 3 O 4	0.074	866.13206	-4.91	-5.25	-4.25	37.50	ok	even
C 49 H 18 F 10 N 5	0.075	866.13970	3.92	3.29	3.40	38.50	ok	even
C 40 H 28 F 13 Ga 1 N 3	0.294	866.13260	-4.28	-5.29	-3.71	22.50	ok	even
C 42 H 29 F 10 Ga 1 N 3 O 2	0.303	866.13504	-1.46	-2.46	-1.27	25.50	ok	even
C 42 H 29 F 6 Ga 1 N 5 O 5	0.310	866.13232	-4.60	-5.63	-3.99	28.50	ok	even
C 44 H 30 F 7 Ga 1 N 3 O 4	0.314	866.13749	1.36	0.37	1.18	28.50	ok	even
C 45 H 28 F 9 Ga 1 N 3 O 1	0.314	866.13390	-2.78	-3.77	-2.41	29.50	ok	even
C 47 H 29 F 6 Ga 1 N 3 O 3	0.325	866.13634	0.04	-0.93	0.04	32.50	ok	even
C 48 H 27 F 8 Ga 1 N 3	0.326	866.13276	-4.10	-5.07	-3.55	33.50	ok	even
C 49 H 30 F 3 Ga 1 N 3 O 5	0.337	866.13879	2.86	1.90	2.48	35.50	ok	even
C 50 H 28 F 5 Ga 1 N 3 O 2	0.338	866.13520	-1.28	-2.24	-1.11	36.50	ok	even
C 50 H 28 F 1 Ga 1 N 5 O 5	0.345	866.13248	-4.42	-5.40	-3.83	39.50	ok	even

Figure S18 – HRMS-ESI(+) spectrum of compound **9**

4. Enthalpy PM7 semi-empirical calculations

Table S1 – Calculations of the formation enthalpies for precursor **8**.

**Compounds**	**Formation enthalpy (kcal mol^{-1})$^{a)}$**	**Formation enthalpy (kcal mol^{-1})$^{b)}$**
**8**	-416.69	-421.36

$^{a)}$ Without solvent. $^{b)}$ in toluene.

Table S2 – Structures and calculations of formation enthalpies for compound **9** tautomers (**9T1**, **9T2** and **9T3**) and structure for tautomer **9T1** optimized with MOPAC2016.

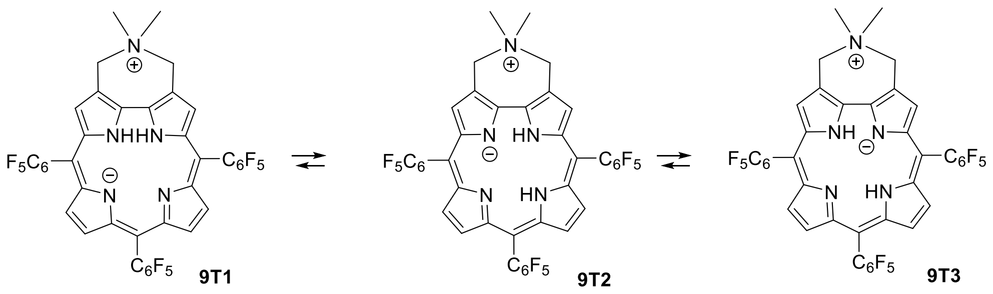

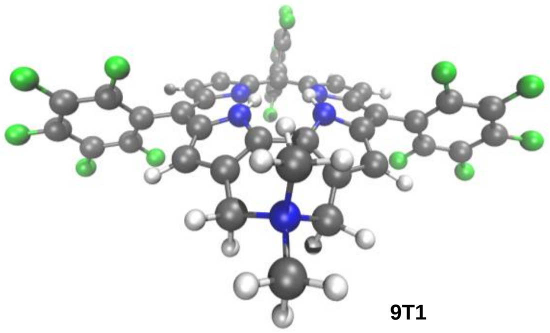

**Compounds**	**Formation enthalpy (kcal mol^{-1})$^{a)}$**	**Formation enthalpy (kcal mol^{-1})$^{b)}$**
**9T1**	-366.54	-380.95
**9T2**	-354.94	-367.21
**9T3**	-361.04	-374.73

$^{a)}$ Without solvent. $^{b)}$ in toluene.

5. Spectophotometric interactions

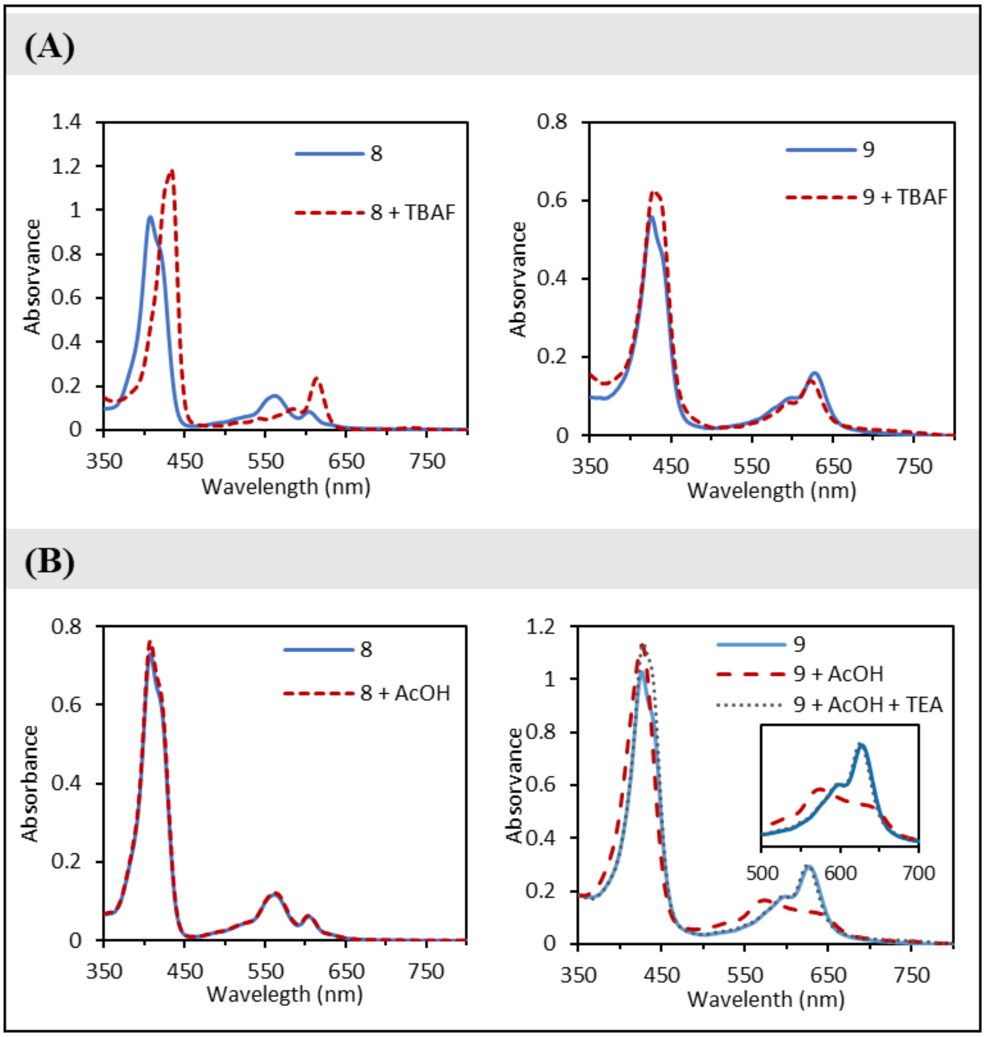

Figure S19 – Absorption spectra of behaviour of corroles **8** and **9** in the presence of **(A)** tetrabutylammonium fluoride (TBAF) and **(B)** acetic acid (AcOH) and AcOH plus triethylamine (TEA).
